# Diagnosis and management of mixed *Chlamydia abortus and psittaci* pneumonia guided by metagenomic next-generation sequencing: a case report

**DOI:** 10.1186/s12879-026-13691-y

**Published:** 2026-05-26

**Authors:** Lin Liu, Pan Su, Fan Gong, Aimin Wang, Xiaobin Wang, Lin Yang, Weisong Mo, Tao Jiang

**Affiliations:** 1 Department of Emergency Medicine, Hunan University of Medicine General Hospital, No. 144 South Jinxi Road, Hecheng District, Huaihua, Hunan Province 418000 China; 2 Department of Emergency Medicine, Xiangya Hospital, Central South University, Changsha, Hunan Province 410008 China

**Keywords:** *Chlamydia abortus*, *Chlamydia psittaci*, Mixed infection, Metagenomic next-generation sequencing, Zoonosis, Doxycycline

## Abstract

**Background:**

*Chlamydia abortus p*rimarily causes abortion and stillbirth in animals and is associated with pregnancy-related complications in humans. However, it is an extremely rare cause of pneumonia in humans. While *Chlamydia psittaci* is a well-established respiratory pathogen, pneumonia resulting from a co-infection with both species has not been previously reported.

**Case presentation:**

A 57-year-old male presented with fever, cough, and shortness of breath. Imaging revealed extensive pulmonary inflammation and consolidation, which rapidly progressed to respiratory failure. Metagenomic next-generation sequencing (mNGS) of bronchoalveolar lavage fluid (BALF) confirmed a mixed infection with *Chlamydia abortus* and *Chlamydia psittaci*. Following the early initiation of targeted doxycycline therapy, the patient’s clinical symptoms and pulmonary imaging showed significant improvement, leading to a full recovery and hospital discharge.

**Conclusions:**

To our knowledge, this study reports the first case of atypical pneumonia caused by a mixed *Chlamydia abortus* and *Chlamydia psittaci* infection in a male patient, thereby expanding the clinical spectrum of these zoonotic pathogens. The case exhibited a “clinical-imaging dissociation,” characterized by severe radiographic changes alongside relatively mild clinical symptoms. When conventional diagnostic methods failed to identify the pathogens, mNGS provided a rapid and precise diagnosis. Guided by this result, early targeted therapy with doxycycline achieved a marked therapeutic effect, preventing progression to severe disease and an adverse outcome.

**Trial registration:**

Not applicable.

## Background

Chlamydiae are a group of obligate intracellular, Gram-negative bacteria widely distributed in nature, capable of infecting insects, birds, mammals, and humans [1, 2]. Over ten species have been identified, with *Chlamydia psittaci*, *Chlamydia trachomatis*, and *Chlamydia pneumoniae* being most clinically relevant to human infections [3]. Notably, birds and mammals serve as reservoir hosts and are primary sources for several zoonotic chlamydial species.

Among pathogenic chlamydiae, *Chlamydia abortus* and *Chlamydia psittaci* both possess significant zoonotic potential but exhibit distinct epidemiological profiles. *Chlamydia abortus* is primarily a pathogen of ruminants (e.g., sheep, goats). Human transmission typically occurs through contact with contaminated materials like aborted fetuses or placentas, potentially leading to severe complications such as abortion and sepsis, particularly in pregnant women, with pneumonia being a rare manifestation [4–7]. In contrast, *Chlamydia psittaci* has birds as its primary reservoir. Human infection usually results from inhaling dust aerosols contaminated by infected birds or their droppings, with a clinical spectrum ranging from mild influenza-like illness to severe pneumonia requiring hospitalization [2].

This study reports a case of severe pneumonia caused by co-infection with *Chlamydia abortus* and *Chlamydia psittaci* in a middle-aged male, rapidly diagnosed via metagenomic next-generation sequencing (mNGS) of bronchoalveolar lavage fluid (BALF). We aim to discuss the clinical characteristics, diagnostic strategy, and therapeutic response in this case, analyze potential infection routes in light of recent epidemiological insights, and thereby provide a reference for managing similar rare infections.

## Case presentation

A 57-year-old male was admitted to our hospital with a 6-day history of fever, cough, and shortness of breath. He reported a maximum body temperature of 40 °C, experiencing 2–4 febrile episodes daily accompanied by headache. He had scant expectoration of yellowish, thin sputum. He felt short of breath upon exertion. Due to inadequate response to treatment at a local hospital, he was transferred to our facility. The patient’s past medical history was unremarkable. He worked as a farmer, raising only pigs at home, which showed no signs of miscarriage or abortion. He had no contact with other livestock or birds.

**Fig. 1:**
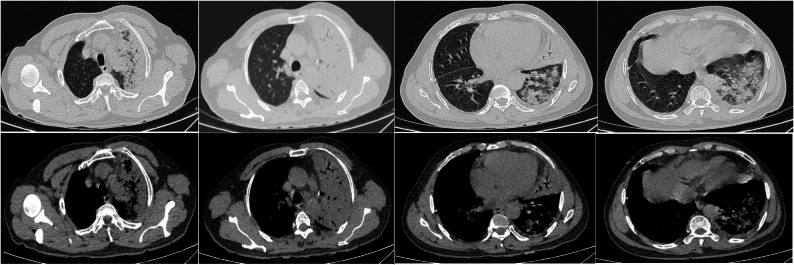
Chest CT on admission shows extensive consolidation and atelectasis in the left upper lobe, with bilateral infiltrates and a small left pleural effusion

On admission to the emergency department, physical examination revealed: body temperature 39.5 °C, pulse 120 beats/min, respiratory rate 25 breaths/min, blood pressure 120/70 mmHg, and peripheral oxygen saturation (SpO_2_) 88% on room air. Coarse breath sounds and scattered moist rales were audible in both lung fields. Initial laboratory tests and arterial blood gas analysis (Table 1) showed: oxygen saturation 92.8%, pH 7.508, partial pressure of carbon dioxide (PaCO_2_) 23.8 mmHg, partial pressure of oxygen (PaO_2_) 58.2 mmHg, actual bicarbonate (HCO_3_^−^-act) 22.3 mmol/L, lactate 1.38 mmol/L, PaO_2_/FiO_2_ ratio 200.69, and fraction of inspired oxygen (FiO_2_) 29.0%. Other results included a white blood cell count of 6.1 × 10⁹/L, neutrophil percentage 89.5%, Lymphocyte percentage 8.3%, eosinophil percentage 0.1%, C-reactive protein (CRP) 150.01 mg/L, procalcitonin (PCT) 2.01 µg/L, fibrinogen 9.26 g/L, and plasma D-dimer 1.92 mg/L. Chest CT (Computed Tomography. Fig. 1) revealed: (1) multiple areas of infection in both lungs, more prominent in the left lung, with consolidation and atelectasis in the left upper lobe; and (2) bilateral pleural thickening and a small amount of left pleural effusion. Oxygen therapy was initiated via nasal cannula at 5 L/min and empirical intravenous antibiotic therapy with piperacillin-tazobactam (4.5 g every 8 h).

**Table 1:** Laboratory test results

Item	Day 1	Day 5	Day 11	Reference Range
PO_2_/FiO_2_	200.69	ND	410.15	> 400
WBC(×10⁹/L)	6.1	4.5	4.9	3.5–9.5
CRP (mg/L)	150.01	55.29	3.21	0–8
PCT (µg/L)	2.01	0.26	ND	0−0.5

Abbreviations: PO_2_/FiO_2_, Partial pressure of oxygen/Fraction of inspired oxygen; WBC, White blood cell count; PCT, Procalcitonin; CRP, C-reactive protein; ND, Not detected

On the second day, the patient was admitted to the Department of Respiratory Medicine for inpatient care. Empirical anti-infective therapy was continued with intravenous piperacillin-tazobactam (4.5 g every 8 h) in combination with doxycycline (100 mg every 12 h). A bronchoscopy with bronchoalveolar lavage (BAL) was performed and the sample obtained was sent for analysis. The results of acid-fast staining, Mycobacterium tuberculosis complex (MTB) DNA testing with rifampin resistance detection, BALF galactomannan (GM) test, and BALF culture were all normal. Metagenomic next-generation sequencing (mNGS) of the BALF (performed by Saizhe Medical Laboratory in Changsha, China) detected *Chlamydia abortus* with 60 sequence reads (relative abundance 32.432%) and *Chlamydia psittaci* with 27 sequence reads (relative abundance 14.595%) (Table 2). Tests for *Chlamydia pneumoniae* IgM, *Mycoplasmoides pneumoniae* IgM, (1,3)-β-D-glucan (G test), and blood cultures were all negative. The combined intravenous antibiotic therapy with piperacillin-tazobatam and doxycycline was maintained.

**Table 2:** mNGS results of BALF

Type	Genus	Species	Sequence Reads	Relative Abundance
P	*Chlamydia*	*Chlamydia abortus*	60	32.432%
P	*Chlamydia*	*Chlamydia psittaci*	27	14.595%

**Fig. 2:**
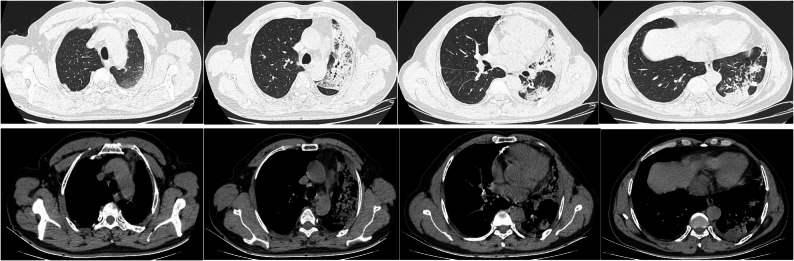
Follow-up chest CT on hospital day 10 demonstrates marked resolution of the consolidation and atelectasis in the left upper lobe

On the fifth day of hospitalization, the patient became afebrile. Repeat blood tests showed normalization of the white blood cell count and procalcitonin (PCT) level, with a significant decrease in C-reactive protein (CRP) (Table 1). A follow-up chest CT scan on the tenth hospital day (Fig. 2) revealed a reduction in the infectious lesions in the left upper lobe and improvement in the consolidation and atelectasis. The left pleural effusion remained essentially unchanged. The patient was free of fever and shortness of breath, with a normal oxygenation index. Following this clinical improvement, he was discharged from the hospital in good condition.

**Fig. 3:**
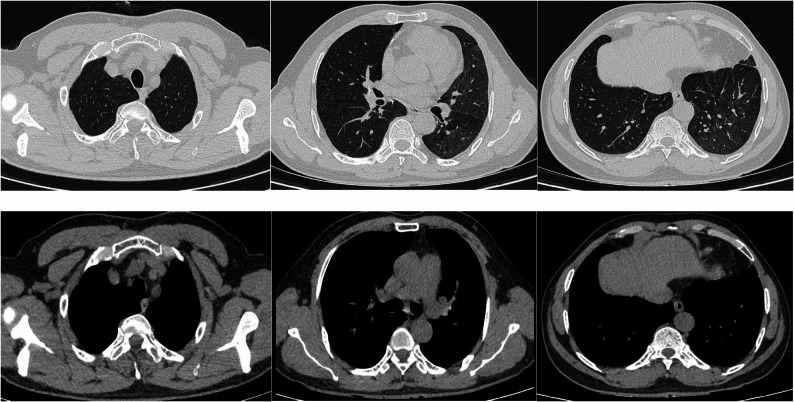
Chest CT one month after discharge reveals near-complete resolution of the pulmonary infiltrates and pleural effusion

Following discharge, the patient continued anti-infective therapy with oral doxycycline capsules (100 mg twice daily) for a total course of two weeks. A follow-up chest CT scan one month after discharge (Fig. 3) showed: (1) significant absorption and reduction of the multiple pulmonary infections compared to previous imaging; and (2) marked improvement in bilateral pleural thickening, with the small left pleural effusion having essentially resolved. A telephone follow-up three months later revealed the patient had no specific complaints and no recurrence of illness.

## Discussion

*Chlamydia abortus* is a zoonotic pathogen closely related to *Chlamydia psittaci* and was historically classified as a subtype of the latter [8, 9]. Species identification was performed using metagenomic sequencing by aligning reads to the reference sequences of *Chlamydia abortus* and *Chlamydia psittaci*. Unique reads mapping to each reference genome were obtained, and 10 reads from each species were selected for BLAST alignment against the RefSeq reference genome database. The specificity of read mapping ensured accurate species differentiation.

To strengthen the causal inference, we interpreted the mNGS results in the context of the patient’s clinical presentation, imaging findings, and therapeutic response. Both *Chlamydia abortus* and *Chlamydia psittaci* were detected with unique reads specifically mapping to their respective reference genomes. Mycobacterium tuberculosis complex (MTB) DNA testing, BALF galactomannan (GM) test, and BALF culture were all normal. Tests for *Chlamydia pneumoniae* IgM, *Mycoplasmoides pneumoniae* IgM, (1,3)-β-D-glucan (G test), and blood cultures were all negative, effectively ruling out other common infectious etiologies. Furthermore, the patient’s prompt clinical and radiological improvement following doxycycline therapy—an antibiotic with established efficacy against Chlamydia species—supports the pathogenic role of both organisms. While the possibility of colonization or contamination cannot be entirely excluded, the constellation of clinical, microbiological, and therapeutic evidence favors a genuine coinfection.

According to published literature, only slightly more than ten confirmed cases of human pneumonia caused by *Chlamydia abortus* infection have been reported globally (Table 3). Transmission typically stems from direct contact with infected animals or a contaminated environment, while the risk associated with indirect contact (e.g., residing near or visiting an infected farm) is relatively low [10, 11]. In the present case, the patient worked as a farmer and raised pigs at home, but reported no direct contact with sheep, goats, poultry, or birds. None of his pigs showed signs of miscarriage or abortion. However, the patient lived in a rural area where livestock farming is common among neighbors. It is possible that indirect environmental exposure—such as inhalation of contaminated dust or aerosols from nearby farms—served as the route of transmission. Although the exact source of infection could not be definitively identified, this case highlights the potential for zoonotic Chlamydia infections even in the absence of direct animal contact. Future epidemiological investigations should consider environmental sampling and serological screening of household members and livestock to clarify transmission dynamics.

This case exhibited a marked “clinical-imaging dissociation,” characterized by extensive consolidation and rapid progression on chest CT alongside relatively limited systemic symptoms. We hypothesize that this phenomenon may be attributable to the unique obligate intracellular life cycle of Chlamydia species. Pathogens replicate extensively within alveolar epithelial cells and macrophages, inciting a robust local cellular immune response and an intense release of inflammatory cytokines (e.g., involving TNF-α and IL-6), which results in severe pulmonary tissue damage while potentially lacking significant bacteremia or systemic toxin release. Furthermore, mixed infection may have further exacerbated the localized inflammatory response [2].

Reports of chlamydial coinfection in the literature are extremely rare, and to our knowledge, this is the first reported case of coinfection with *Chlamydia abortus* and *Chlamydia psittaci*. Mixed infection may potentiate local tissue damage through synergistic immune evasion or competition for the same intracellular niche [2]. Theoretically, dual infection carries a risk of genetic recombination, although no evidence of such recombination was identified in this study. Future investigations using whole-genome deep sequencing of clinical samples are warranted to further explore this possibility. Nevertheless, the precise underlying mechanisms remain to be elucidated. Clinicians should maintain a high index of suspicion for uncommon pathogens when encountering pneumonia characterized by severe radiographic findings but atypical initial systemic toxicity symptoms.

The definitive diagnosis in this case was critically dependent on metagenomic next-generation sequencing (mNGS) of bronchoalveolar lavage fluid (BALF). Compared to conventional diagnostic methods, mNGS offered notable advantages. First, its hypothesis-free, unbiased approach overcomes the limitations imposed by clinical preconceptions. Following negative results from conventional bacterial cultures, Mycoplasma/Chlamydia serology, and targeted PCR, mNGS successfully identified the rare pathogens, thereby averting diagnostic delay and potentially unnecessary antibiotic use [2]. Second, its high-throughput capability directly revealed the co-infection with both *Chlamydia abortus* and *Chlamydia psittaci*—a finding that would be challenging and less efficient to obtain through traditional, sequential pathogen-specific tests. These core strengths make mNGS particularly suitable for diagnosing novel, rare, mixed, or refractory infections in patients who have already received empirical antimicrobial therapy [12, 13]. It is important, however, to acknowledge its limitations objectively. The interpretation of mNGS results is heavily reliant on the comprehensiveness of genomic databases, and the detection of microbial nucleic acids does not invariably signify viable, causative organisms. Although the sequence reads obtained in our study were relatively low in number, the causality was supported by the classic clinical presentation and the patient’s prompt response to doxycycline. Notably, this case, along with the majority of domestically reported *Chlamydia abortus* pneumonia cases in recent years (Table 3) [14–20], was ultimately confirmed by BALF-based mNGS after conventional testing yielded negative results. This highlights the pivotal role of mNGS in resolving diagnostic dilemmas in pneumonia of unknown etiology, particularly those involving zoonotic pathogens.

The accurate etiological diagnosis provided by mNGS paved the way for targeted antimicrobial therapy. Regarding antimicrobial therapy, chlamydiae are generally susceptible to antibiotics that inhibit protein synthesis by targeting the bacterial ribosome, such as tetracyclines, macrolides, and quinolones [21]. As no dedicated treatment guidelines exist for *Chlamydia abortus* infection, clinical management typically follows recommendations for *Chlamydia psittaci.* Based on current evidence and consensus guidelines, tetracycline-class antibiotics, particularly doxycycline, are recommended as first-line agents due to their demonstrated efficacy against various zoonotic chlamydial infections. For severe cases, initial intravenous administration is crucial to achieve adequate plasma drug concentrations [22, 23]. Macrolides (e.g., azithromycin) serve as important alternatives, especially for patients with contraindications to tetracyclines. Although quinolones possess in vitro activity, clinical efficacy data remain relatively limited, and they are generally not considered first-choice agents [22, 24].

The successful outcome in our case reinforces the role of doxycycline as a first-line therapeutic agent for zoonotic chlamydial pneumonia. A review of the cases summarized in Table 3 suggests that the critical determinants of treatment success or failure may not be the specific antimicrobial agent alone. The timing of therapy initiation and the management of severe complications appear to be paramount. Both fatal cases (Cases 7 and 13), which progressed rapidly to acute respiratory distress syndrome (ARDS) and multiple organ dysfunction syndrome (MODS) requiring extracorporeal membrane oxygenation (ECMO) support, occurred despite the administration of doxycycline or a novel tetracycline (omadacycline). This indicates that once a dysregulated systemic inflammatory response and multi-organ failure are established, even pathogen-directed antibiotics may be insufficient to reverse the outcome. In contrast, most surviving patients received effective treatment prior to the development of severe respiratory failure. In our patient, doxycycline was added empirically on the second hospital day, preceding the return of mNGS results, which likely secured a critical window for early intervention. This experience highlights a key clinical practice point: in cases of suspected zoonotic atypical pneumonia—especially when an epidemiological link to animals is present or when severe imaging findings contrast with milder systemic symptoms—empirical tetracycline therapy should be initiated without delay, rather than awaiting microbiological confirmation.

**Table 3:** Summary of reported *Chlamydia abortus* infection cases

No.	Year	Country	Age/Sex	Animal Contact History	Complications	Life Support	Diagnostic Method	Time to Diagnosis	Treatment	Outcome	Reference
1	2016	Spain	47/M	Veterinarian	None	Nasal cannula	ELISA serology	8 days	Clarithromycin	Recovery	[25]
2	2020	France	27/F (Pregnant)	Sheep	Shock, ARDS	Invasive ventilation	Placental PCR	NR	Doxycycline	Recovery	[5]
3	2022	Switzerland	34/F (Pregnant)	Goats	Severe Pneumonia, Septic Shock, ARDS	NR (Likely nasal cannula)	Throat swab PCR	6 days	Clarithromycin	Recovery	[26]
4	2022	China	66/M	NR	Bloodstream infection, Respiratory Failure	Nasal cannula	BALF-mNGS	NR	Moxifloxacin	Recovery	[14]
5	2022	China	65/M	No direct contact	Bloodstream infection, Septic Shock, Respiratory Failure, MODS	NR	BALF& Blood mNGS	NR	Azithromycin, Levofloxacin	Recovery	[15]
6	2023	China	51/M	Poultry	Hypoxemia	Nasal cannula	BALF-mNGS	NR	Doxycycline	Recovery	[8]
7	2023	China	44/M	Denied contact	ARDS, Sepsis, MODS	ECMO, Invasive ventilation, Prone position, Neuromuscular blockade, CRRT	BALF-mNGS	8 days	Doxycycline, Azithromycin	Death	[11]
8	2024	China	74/F	Poultry	Severe Pneumonia	Invasive ventilation	BALF-mNGS	15 days	Doxycycline, Moxifloxacin	Recovery	[16]
9	2024	China	60/F	Poultry	Severe respiratory distress, Abnormal liver function	Invasive ventilation	BALF-mNGS	17 days	Levofloxacin, Omadacycline	Recovery	[17]
10	2024	China	76/M	Poultry	ARDS, Sepsis	Invasive ventilation, Prone position	BALF-mNGS	9 days	Moxifloxacin	Recovery	[18]
11	2024	China	48/M	Birds	None	Nasal cannula	BALF-mNGS	9 days	Azithromycin	Recovery	[19]
12	2025	Netherlands	74/M	Wild waterfowl	Severe Pneumonia	Invasive ventilation	Sequencing (non-mNGS)	NR	Doxycycline	Recovery	[27]
13	2025	China	42/F	Denied contact	ARDS, MODS, Hemophagocytic syndrome	ECMO, Invasive ventilation, Prone position	BALF-mNGS	10 days	Omadacycline	Death	[20]

## Conclusion

This study reports the documented case of atypical pneumonia caused by a mixed infection of *Chlamydia abortus* and *Chlamydia psittaci* in a male patient, which presented with a clinical-imaging dissociation phenotype. Confronting the diagnostic limitations of conventional methods, metagenomic next-generation sequencing (mNGS) enabled rapid and precise pathogen identification, guiding early doxycycline therapy that yielded a significant clinical response. This case underscores the importance of integrating mNGS into the early diagnostic workup for complex pneumonia to facilitate the timely detection of uncommon pathogens and guide targeted treatment. Future research should further elucidate the transmission and pathogenesis of such infections and develop diagnostic models that incorporate novel technologies, with the ultimate goal of formulating evidence-based preventive guidelines and individualized management strategies for the early recognition and precise management of these zoonotic respiratory infections.

## Data Availability

The raw sequence data reported in this paper have been deposited in the Genome Sequence Archive (Genomics, Proteomics & Bioinformatics 2025) in National Genomics Data Center (Nucleic Acids Res 2025), China National Center for Bioinformation / Beijing Institute of Genomics, Chinese Academy of Sciences (GSA-Human: HRA016419) that are publicly accessible at https://ngdc.cncb.ac.cn/gsa-human. All other clinical data supporting the findings of this study are included within this article and its supplementary files.

## Abbreviations

ARDS: Acute Respiratory Distress Syndrome
BALF: Bronchoalveolar Lavage Fluid
CRRT: Continuous Renal Replacement Therapy
ECMO: Extracorporeal Membrane Oxygenation
ELISA: Enzyme-Linked Immunosorbent Assay
mNGS: Metagenomic Next-Generation Sequencing
MODS: Multiple Organ Dysfunction Syndrome
NR: Not Reported
PCR: Polymerase Chain Reaction
